# Tonsillar Post-transplant Lymphoproliferative Disorder Presenting With Hemoptysis After Heart Transplantation: A Case Report and Review of the Literature

**DOI:** 10.7759/cureus.107419

**Published:** 2026-04-20

**Authors:** Jacob Surma, Theresa A Schneider, Jordann Cress, Edward J Krowiak

**Affiliations:** 1 Otolaryngology, Covenant HealthCare College of Medicine at Central Michigan University, Mount Pleasant, USA; 2 Otolaryngology-Head and Neck Surgery, Henry Ford Hospital, Warren, USA; 3 Otolaryngology-Head and Neck Surgery, Ascension St. Vincent Hospital, Indianapolis, USA

**Keywords:** cytomegalovirus co-infection, epstein-barr virus, heart transplantation, hemoptysis, polymorphic post-transplant lymphoproliferative disorder, tonsillar necrosis, tonsillar ptld, tonsillectomy, waldeyer's ring

## Abstract

Post-transplant lymphoproliferative disorder (PTLD) is a potentially fatal complication of solid organ transplantation. Heart transplant recipients are among those at elevated risk for developing PTLD. Tonsillar involvement is a recognized manifestation, typically presenting as recurrent tonsillitis or progressive tonsillar hypertrophy, although hemorrhagic presentations are rare.

We present the case of a 44-year-old man with nonischemic cardiomyopathy, status post-orthotopic heart transplantation in December 2024, who developed recurrent tonsillitis with progressive bilateral tonsillar hypertrophy in the setting of active Epstein-Barr virus (EBV) and cytomegalovirus (CMV) co-infection. He initially presented with a four-month history of throat pain and was subsequently found to have epiglottitis, for which he was treated with antibiotics, antivirals, and corticosteroids. Two months later, he presented to the emergency department (ED) with hemoptysis after a coughing episode, reporting expulsion of a large blood clot. Laboratory studies revealed a hemoglobin (Hgb) of 6.6 g/dL and leukopenia, necessitating transfusion of packed red blood cells (pRBCs). Flexible fiberoptic laryngoscopy (FFL) demonstrated blood pooling in the supraglottis, suspected to originate from the palatine tonsils. Over the following days, he developed a perforation of the right anterior tonsillar pillar with tonsillar necrosis and required additional transfusion for persistent anemia. Bilateral tonsillectomy with direct laryngoscopy was performed, revealing necrotic tonsillar tissue with sloughing extending to the lateral pharyngeal walls such that normal tissue planes were difficult to establish. Following tonsillectomy, hemoptysis resolved completely, Hgb levels stabilized, and no further transfusions were required. Final surgical pathology was consistent with polymorphic PTLD, supported by fluorescence in situ hybridization demonstrating loss of BCL6/3q27, MYC/8q, BCL2/18q21.3, and IGH/14q32. Flow cytometry was negative for overt lymphoma.

A systematic literature search of PubMed/MEDLINE across 10 thematic areas identified 131 unique articles. Combined with a bibliography review, 30 references were selected for inclusion. A review of the literature revealed no previous reports of hemoptysis originating from tonsillar PTLD or resolving after tonsillectomy for PTLD. To our knowledge, this is the first reported case of hemoptysis as a presenting feature of tonsillar PTLD in an adult heart transplant recipient. This case emphasizes the importance of maintaining a high index of suspicion for PTLD in transplant recipients with persistent or progressive tonsillar disease accompanied by hemorrhagic or necrotic features, and reinforces the dual diagnostic and therapeutic role of tonsillectomy in the management of localized tonsillar PTLD.

## Introduction

Post-transplant lymphoproliferative disorder (PTLD) is a potentially fatal complication following solid organ transplant (SOT) or hematopoietic stem cell transplantation, leading to excessive B cell proliferation within a weakened immune system [1,2]. Ranging from benign growth to malignant lymphoma, the incidence of PTLD is estimated to be 0.5-1% with SOT recipients at a 50-120% greater risk [3].

One of the largest risk factors for developing PTLD is infection with the Epstein-Barr Virus (EBV). This is partially due to EBV’s ability to alter and immortalize B lymphocytes; however, the full pathogenesis is complex [4]. The core mechanism involves supratherapeutic immune suppression, allowing viral-induced unchecked proliferation of lymphoid tissues [5]. With excessive suppression, other viruses, such as cytomegalovirus (CMV), present an additional risk for the development of PTLD. Often co-infected, patients with dual EBV and CMV seropositivity are associated with more serious outcomes, including graft rejection and heightened malignant PTLD potential, with PTLD rates reported as high as 92-100% among co-infected patients [6]. This may be partly explained by CMV-mediated depletion of natural killer (NK) cell subsets critical for EBV immune surveillance [7]. 

Lymphoid tissues most commonly affected by PTLD include the gastrointestinal tract, lymph nodes, Waldeyer's ring (including the tonsils and adenoids), liver, and lungs, with extranodal involvement reported in the majority of cases [8]. Among heart transplant recipients specifically, the largest registry analysis to date found that 3.8% developed PTLD within 10 years of transplantation, with PTLD development within three years conferring a significant mortality risk [9]. Within this population, 39% were present in the head and neck [10]. Tonsillar involvement is a well-recognized manifestation of PTLD in this region, often presenting as recurrent or refractory tonsillitis, progressive tonsillar hypertrophy, or obstructive symptoms in transplant recipients [11,12]. Acute nonbacterial tonsillitis may be the earliest clinical sign, particularly in the setting of active EBV infection [13]. Tonsillectomy in these patients serves a dual diagnostic and therapeutic role, with PTLD identified in up to 47% of tonsillectomy specimens from transplant recipients [14]. Notably, destructive tonsillar features, including necrosis, ulceration, and fistulation, have been reported, although rarely [15,16]. Hemorrhagic manifestations of PTLD, including epistaxis and oropharyngeal bleeding, are rare, with hemoptysis from tonsillar PTLD previously unreported in the literature [17,18]. 

Here, we present the case of a 44-year-old man, a heart transplant recipient with EBV and CMV co-positivity, who developed recurrent tonsillitis with progressive tonsillar hypertrophy, anterior pillar perforation, transfusion-dependent anemia, and hemoptysis, ultimately diagnosed with PTLD on tonsillectomy pathology. To our knowledge, this is the first reported case of hemoptysis originating from tonsillar PTLD in an adult heart transplant recipient.

## Case presentation

A 44-year-old man with a history of nonischemic cardiomyopathy and nonsustained ventricular tachycardia, status post Abbott HeartMate 3 (Abbott Laboratories; Abbott Park, IL, USA) left ventricular assist device (LVAD) placement in 2019, underwent orthotopic heart transplantation in December 2024. His post-transplant course was complicated by acute kidney injury (AKI) requiring continuous venovenous hemofiltration, with eventual renal recovery. He additionally developed a persistent right groin wound necessitating incision and drainage with wound vacuum-assisted closure placement. There was no history of allograft rejection. He had no previous history of tobacco, alcohol, or illicit drug use.

The patient first presented to the emergency department (ED) on November 28, 2025, with a four-month history of throat pain and otalgia. He reported dysphagia associated with odynophagia. Computed tomography (CT) of the neck demonstrated thickening of the aryepiglottic folds and posterior wall of the hypopharynx with airway narrowing; however, no discrete mass was identified. Both the group A *Streptococcus pyogenes* rapid antigen test and mononucleosis screen were negative.

He subsequently presented to the otolaryngology clinic on December 1, 2025. On examination, the tonsils were bilaterally hypertrophied at 3+ and symmetric. Flexible fiberoptic laryngoscopy (FFL) demonstrated circumferential redundant pharyngeal tissue without obstruction or a discrete mass. The palatine tonsils were enlarged with exudates. Aerobic and fungal cultures were obtained at the visit. Ten days later, he presented to the ED with worsening throat pain and was admitted on December 11, 2025. He was found to have epiglottitis in the setting of active EBV and CMV co-infection. Otolaryngology evaluation revealed stable tonsillar enlargement at 3+, and infectious disease consultation was obtained. The patient was started on ceftriaxone 2 g intravenously twice daily (BID), azithromycin 500 mg orally once daily, valganciclovir 450 mg orally BID, and intravenous (IV) dexamethasone 10 mg every eight hours for a total of three doses. The transplant team adjusted his immunosuppressive regimen during this admission. Gastroenterology performed an esophagogastroduodenoscopy (EGD), which demonstrated gastritis with evidence of bleeding; a hemostatic agent was applied (specific agent not specified in operative note). This admission was further complicated by an AKI with a peak creatinine of five, which trended downward by discharge. The patient was discharged on December 22, 2025, after an 11-day hospitalization and was maintained on oral valganciclovir.

On February 1, 2026, approximately six weeks after discharge, the patient presented to the ED with hemoptysis following a significant coughing episode at home. He reported that his throat pain from the previous admission had initially improved but had begun to worsen again two days before presentation. He had experienced several coughing episodes in the days preceding the hemoptysis event. Before arrival at the ED, he recalled expelling a “baseball-sized” blood clot during a coughing episode.

On arrival, his vital signs were notable for a temperature of 97.8°F, heart rate of 126 beats per minute, respiratory rate of 18 breaths per minute, blood pressure of 117/58 mmHg, and peripheral oxygen saturation of 96% on room air. Initial laboratory studies were remarkable for a hemoglobin (Hgb) of 6.6 g/dL, hematocrit of 20.7%, and white blood cell (WBC) count of 1.0 × 10³/µL. One unit of packed red blood cells (pRBCs) was transfused in the ED.

While in the ED, the patient had an additional witnessed episode of hemoptysis, which resolved following administration of 1 g IV tranexamic acid (TXA) and one dose of 500 mg nebulized TXA.

Otolaryngology examination revealed moist mucous membranes with a blood clot overlying the right tonsil. Bright red blood was noted within the oral cavity, and the posterior pharynx was erythematous. The tonsils were bilaterally hypertrophied and graded 3+. An area of mucosal irregularity, consistent with either an abrasion or adherent blood clot, was noted along the oropharynx. Anterior rhinoscopy demonstrated a blood clot within the right nasal cavity without active hemorrhage. FFL revealed blood within the right nasal cavity without an active nasal source, as well as blood pooling in the supraglottis, suspected to originate from the palatine tonsils.

Computed tomography angiography (CTA) of the neck and chest was recommended; however, this could not be obtained due to the patient’s impaired renal function (creatinine of 2.1 mg/dL at the time of admission). The patient was started on cefepime 2 g IV BID and prednisone 5 mg daily. Nebulized TXA was continued on an as-needed basis for recurrent bleeding episodes. At the time of admission, his medication regimen included valganciclovir 450 mg BID, mycophenolic acid 180 mg BID, and tacrolimus 5 mg BID, in addition to medications for diabetes, hypertension, anxiety, and pain management. Admission tacrolimus levels were noted to be supratherapeutic at 28 ng/mL; however, repeat levels the following day measured 4.3 ng/mL, raising concern for a laboratory error. His immunosuppressive regimen was continued without modification.

**Figure 1:**
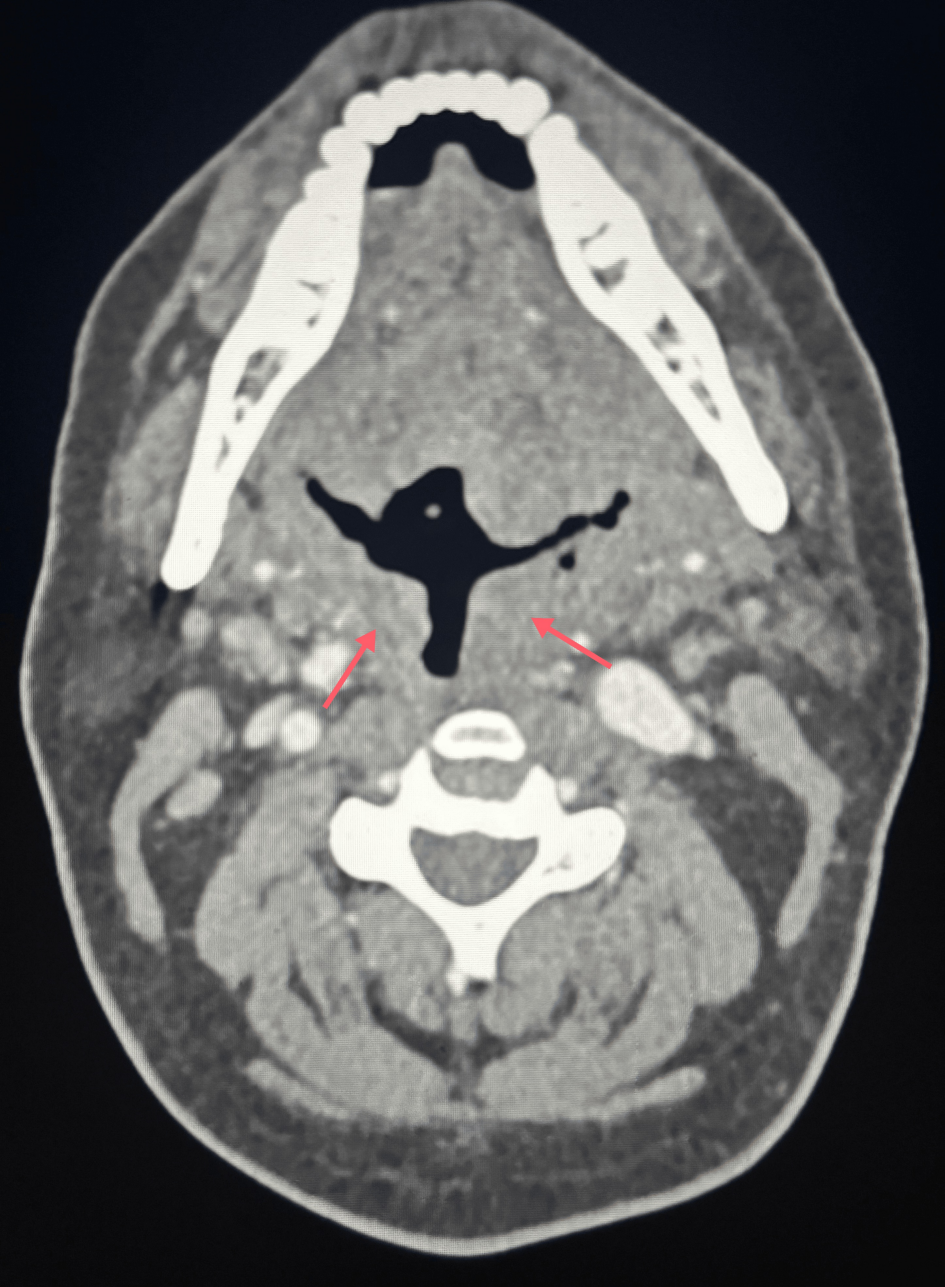
Axial contrast-enhanced CT of the neck demonstrating oropharyngeal soft tissue thickening Axial contrast-enhanced CT image of the neck demonstrating moderate soft tissue thickening along the left oropharynx (red arrows), with involvement of adjacent structures and mild narrowing of the oropharyngeal airway. No discrete source of hemorrhage or rim-enhancing collection is identified. CT: computed tomography

**Figure 2:**
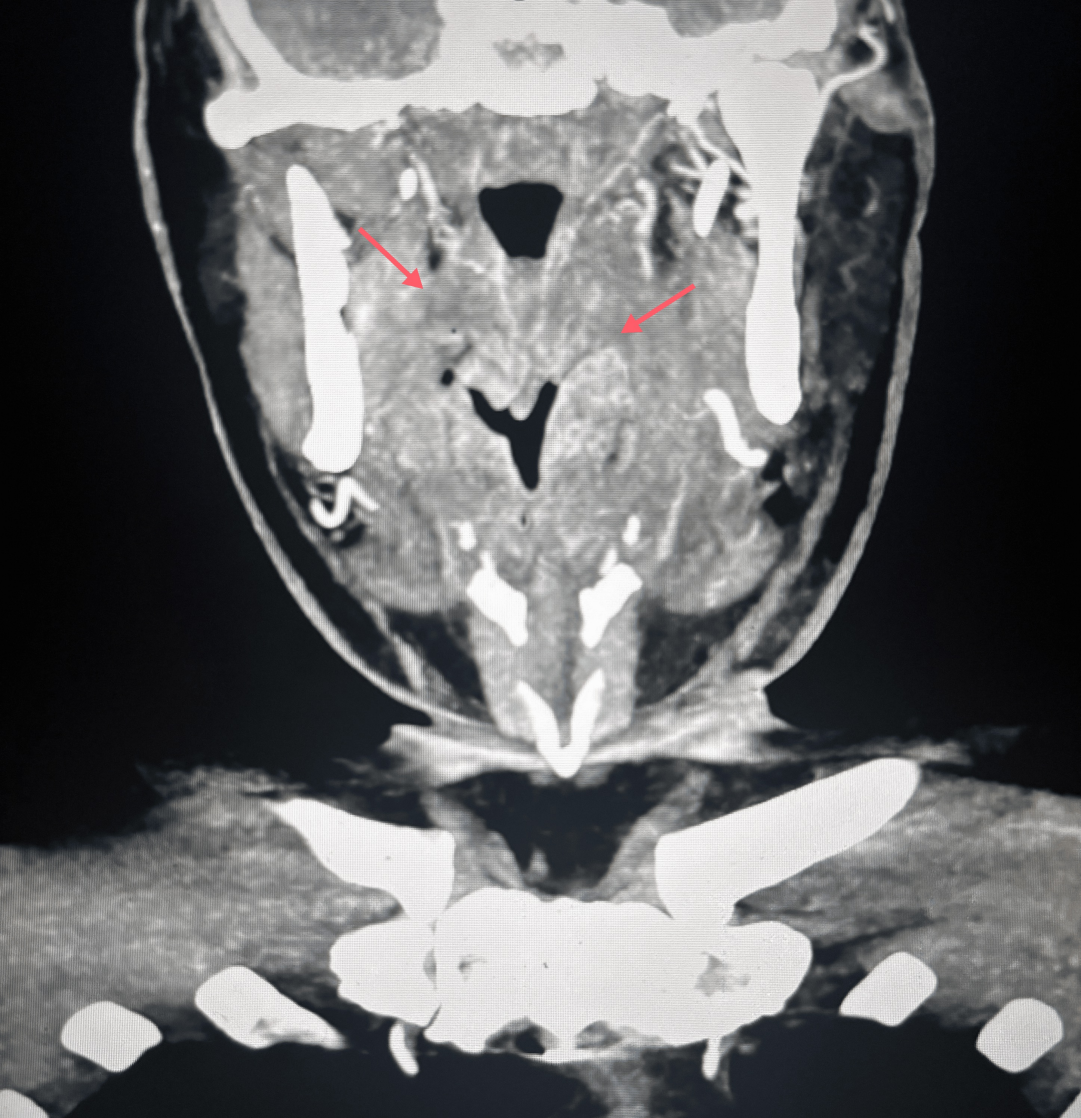
Coronal contrast-enhanced CT of the neck demonstrating oropharyngeal soft tissue thickening Coronal contrast-enhanced CT image of the neck demonstrating asymmetric soft tissue thickening centered in the left oropharynx (red arrows), involving the region of the uvula and epiglottis, with associated airway narrowing. CT: computed tomography

Over the ensuing days, the patient continued to report hemoptysis, although no active source of bleeding was identified on serial examinations. Repeat FFL performed on February 3, 2026, showed no blood within the nasal cavity, oropharynx, or supraglottis, and no definitive bleeding source was visualized. He required an additional transfusion of one unit of pRBCs on this day. CT of the sinuses was obtained and was negative for a source of hemorrhage. CT of the chest with contrast was similarly unremarkable. CT of the neck with contrast demonstrated moderate soft tissue thickening near the left side of the oropharynx involving the uvula and epiglottis (Figures 1, 2).

On February 4, 2026, examination revealed a new finding of a perforation in the right anterior tonsillar pillar, approximately 3 mm in diameter. The right tonsil appeared bruised and possibly necrotic. The tonsils remained bilaterally hypertrophied at 3+. Infectious disease, pulmonology, and gastroenterology services were consulted during the admission. Pulmonology evaluated the patient and determined that bronchoscopy was not indicated. Although gastroenterology was consulted, the service deferred evaluation given the clinical suspicion for an oropharyngeal source of bleeding.

On February 5, 2026, the patient was taken to the operating room, where direct laryngoscopy with biopsy and bilateral tonsillectomy were performed. Intraoperatively, the bilateral tonsils were noted to be necrotic with tissue sloughing. These changes extended to the lateral pharyngeal walls, making the border between the lateral pharyngeal wall and the tonsil difficult to establish. The remaining tonsillar tissue was excised using the standard tonsillectomy technique. Hemostasis was achieved with suction electrocautery, and FloSeal hemostatic matrix (Baxter International Inc., Deerfield, IL, USA) was applied to the tonsillar fossae.

Following tonsillectomy, no further episodes of hemoptysis were observed. Hgb levels trended upward, and no additional blood transfusions were required. Final surgical pathology was consistent with polymorphic PTLD. This was supported by a high-grade lymphoma profile (interphase FISH), which indicated the specimen had a loss of BCL6/3q27, MYC/8q, BCL2/18q21.3, and IGH/14q32, all abnormal results. Flow cytometry was negative for overt lymphoma.

## Discussion

Literature search

A systematic literature search was performed using PubMed/MEDLINE on February 20, 2026, across 10 predefined thematic areas with no date restrictions (inception through February 2026). Articles in all languages were included, provided English-language abstracts were available. Boolean operators were used to combine disease-specific, anatomy-specific, and transplant-specific terms across themes, including tonsillar PTLD, PTLD after heart transplantation, EBV and CMV co-infection, hemorrhagic manifestations of PTLD, necrotizing tonsillar lesions, and tonsillectomy as a diagnostic and therapeutic intervention. Medical subject heading (MeSH) terms were used to supplement keyword searches. A total of 131 unique articles were identified across all thematic searches; reference lists of retrieved articles were hand-searched for additional relevant publications. References were selected based on relevance to the clinical features of the present case, methodological quality, and contribution to the discussion of tonsillar PTLD pathogenesis, presentation, diagnosis, and management, yielding a final reference list of 30 sources. 

Necrotizing tonsillitis and EBV/CMV-associated tonsillar disease

As stated previously, PTLD can manifest in various locations, often with extranodal involvement. As for why the tonsils were targeted in this patient, this is likely due to a multitude of factors, starting with how EBV’s natural lifecycle centers on the tonsil. The virus first enters the body through oropharyngeal epithelium and establishes primary infection within tonsillar subepithelial naive B cells. From this reservoir, EBV drives infected cells through germinal center reactions via successive latency programs, ultimately establishing lifelong inhabitation of memory B cells (which eventually exit the germinal center, explaining extranodal involvement). Thus, the tonsils theoretically harbor the largest reservoir of EBV-infected B cells in the body [19-21]. This pathophysiology is supported by the findings of Torgbor et al., who identified tonsillar marginal zone B cells as the closest cellular precursor to immunosuppression-related B-cell lymphomas [22].

Beyond a logical entry point, Waldeyer’s ring contains a uniquely permissive microenvironment. Lymphoproliferation is favored in this region as the tonsil is a secondary lymphoid organ with active germinal centers. Constant B-cell proliferation, antigen presentation, and cytokine signaling provide the means for EBV to thrive in these tissues. With the addition of CMV co-infection, which depletes NK cell subsets critical for EBV control, the resulting effect was a perfect storm that allowed PTLD to manifest within the tonsillar tissue in this patient [7].

Once established in the oropharynx, PTLD most commonly presents with tonsillar hypertrophy and recurrent tonsillitis, although the clinical presentation has been more extensively characterized in pediatric populations [13]. This patient initially presented with similar findings to those described in pediatric PTLD, with episodes of recurrent tonsillitis and outpatient workup. However, his disease state quickly progressed to include necrotic tonsillar tissue and perforation of the anterior pillar, which represents an unusual and aggressive course. Necrotizing tonsillar features in PTLD have been rarely reported, with Heyes et al. describing one case of erosion of both anterior and posterior pillars in a renal transplant recipient, whereas Spinato et al. documented necrosis found not in tonsillar tissue, but adenoid tissue [15,16]. The etiology of this necrosis likely involves an interplay between rapid lymphoproliferative infiltration outpacing local blood supply, EBV lytic replication within the tonsillar epithelium, and impaired tissue repair in an immunocompromised state. These compounding features are hypothesized to have contributed to the patient’s hemoptysis.

Hemoptysis in post-transplant patients with PTLD

The novelty of this case resides in the presentation of hemoptysis. Only three articles identified from the literature search link hemoptysis/hemorrhage to PTLD, and none involve tonsillar origin. Saha reports a case of massive hemoptysis from pulmonary/endobronchial origin, Clarke et al. revealed epistaxis from nasopharyngeal origin, and Jiang et al. found oropharyngeal hemorrhage requiring tonsillar artery ligation [17,18,23]. Jiang et al. was the closest hemorrhagic comparator; however, the patient did not suffer from hemoptysis specifically.

Based on the available evidence from the patient’s records, the source of hemoptysis likely originated from one of the major vessels supplying the palatine tonsils. The necrotic, friable tonsillar tissue provided the vascular substrate for hemorrhage and could explain the pooling of blood in the supraglottis found on FFL. As the blood passed down along the pharynx and larynx, it likely stimulated mechanosensitive rapidly adapting receptors (RARs) within the laryngeal mucosa, triggering the cough reflex [24]. Laryngeal stimulation is known to produce an immediate expiratory response designed to protect the airway from aspiration, and in this case, from blood. This forceful expulsion would account for the presentation of hemoptysis rather than hematemesis and explains the retrograde passage of blood into the nasopharynx, consistent with the clots found in the right nasal cavity on anterior rhinoscopy.

Treatment of PTLD

Tonsillectomy was the curative treatment for this patient’s hemoptysis. This decision is well documented in the literature and is supported by current guidelines for PTLD treatment [12]. The American Society of Transplantation (AST) Infectious Diseases Community of Practice guidelines note that when surgical excision has been employed for localized disease alongside reduction of immunosuppression (RIS), long-term remission has been observed without the need for additional systemic therapies [25]. Especially for the management of local complications, such as hemorrhage or perforation, surgery remains an essential component of treatment [8,25].

More broadly, the current standard of care for PTLD follows a sequential approach: RIS as initial intervention, rituximab for CD20-positive disease that does not respond, and cytotoxic chemotherapy (rituximab-cyclophosphamide, hydroxydaunorubicin, Oncovin, prednisone (R-CHOP)) reserved for those with disease progression despite rituximab use [25,26]. Unfortunately, the diagnosis of PTLD was not suspected until after pathology returned after tonsillectomy, leading to a slight deviation from the standard of care.

As such, this patient’s clinical course highlights the diagnostic challenge posed by tonsillar PTLD, which can frequently mimic infectious or inflammatory disease in the immunocompromised host. The clinical picture was appropriately managed according to the working diagnoses. Intravenous dexamethasone was administered during the December admission for epiglottitis-associated airway edema, consistent with evidence supporting systemic corticosteroids for reducing supraglottic inflammation, whereas tonsillectomy during the February admission was indicated for refractory hemorrhage [27,28]. Although appropriate for the working diagnoses, corticosteroids carry the potential to further suppress EBV-specific cytotoxic T-lymphocyte responses that are critical for controlling EBV-driven lymphoproliferation [25]. Additionally, immunosuppression was not formally reduced during the February admission. In retrospect, the combination of active EBV and CMV co-infection, progressive tonsillar hypertrophy refractory to antiviral treatment, and the development of necrotic and hemorrhagic features may have warranted earlier consideration of PTLD with a lower threshold for tissue biopsy, consistent with the recommendations of Broughton et al. and Heyes et al. for transplant recipients with persistent tonsillar hypertrophy [12,14].

Comparable cases in the literature 

From the data available, there are no cases that exactly match that of this patient. As stated before, heart transplant recipients manifest PTLD in the head and neck region in approximately 39% of individuals [10]. Despite this frequency, case reports describing tonsillar involvement remain scarce. The targeted search combining heart transplant, tonsil, and PTLD returned only five results. The closest published case to the present report is that of Fairley et al., who described a 32-year-old man, a heart transplant recipient on cyclosporine, who developed a tonsillar and tongue base tumor with EBV-associated lymphoproliferative disease requiring urgent otolaryngologic intervention [29]. However, that case lacked the hemorrhagic, necrotic, and anemic features central to the present patient's course. Aggarwal et al. described a 26-year-old man, a renal transplant recipient with EBV-positive diffuse large B-cell lymphoma. presenting as tonsillar PTLD with acute airway obstruction, but in a different transplant type and without hemorrhagic complications [30]. Jiang et al. described a kidney transplant recipient with oropharyngeal hemorrhage requiring tonsillar artery ligation; this remains the most comparable hemorrhagic case, but involved T/NK-cell rather than B-cell PTLD and did not present with hemoptysis specifically [23].

The present case thus represents a unique convergence of features not previously reported. The implication of these findings can lead to better identification of PTLD symptoms on presentation, as well as add to the body of evidence that supports tonsillectomy as a definitive management option for tonsillar PTLD among adults.

## Conclusions

This case represents the first reported instance of hemoptysis originating from tonsillar PTLD in an adult heart transplant recipient. The combination of recurrent tonsillitis, EBV and CMV co-positivity, progressive tonsillar hypertrophy with anterior pillar perforation and necrosis, transfusion-dependent anemia, and hemoptysis resolving after tonsillectomy constitutes a clinical presentation previously undocumented in the literature. The resolution of hemoptysis following tonsillectomy further reinforces the dual diagnostic and therapeutic role of surgical excision in managing localized tonsillar PTLD.

This case also emphasizes the importance of maintaining a high index of suspicion for PTLD in transplant recipients who present with persistent or progressive tonsillar disease, particularly when accompanied by hemorrhagic or necrotic features refractory to antimicrobial therapy. Clinicians managing patients with SOT should consider early tissue biopsy in the setting of tonsillar hypertrophy that fails to respond to standard treatment, as timely diagnosis of PTLD enables appropriate RIS and initiation of targeted therapy. Further research is needed to characterize hemorrhagic manifestations of tonsillar PTLD and establish evidence-based guidelines for the management of this rare but clinically significant presentation in adult heart transplant recipients.
